# Hernia: Strangulated and Reducible, with Cure by Subcutaneous Injections

**Published:** 1881-06

**Authors:** 


### BIBLIOGRAPHICAL.

HERNIA; Strangulated and Reducible, with Cure by Subcutaneous Injections, Together with Suggested and Improved Methods for Kelotomy. Also an Appendix Giving a Short Account of Various New Surgical Instruments. By Joseph H. Warren, M.D., Member of Am. Med. Ass'n., etc., with Illustrations. Charles N. Thomas, Boston. 1881.

The good results promised by the subcutaneous injection of remedies in the treatment of hernia, if practically realized, heralds an improvement that will prove a blessing of no indifferent magnitude to humanity. Very justly will it supplant the bunglesome and often dangerous endeavors that mark the history of the treatment of this disease from Chauliac to Wood.

It is claimed that to Dr. Joseph Pancoast, of Philadelphia, is due the honor of first using subcutaneous injections in the local treatment of hernia. To the author of the above work is due the praise of having improved this plan of treatment, and of bringing it fully to the consideration of the profession. It would be well for every surgeon to test the truth of the assertions made in the book, and report his results. In this way the claims of the projectors of the plan may be substantiated, or take the course of most of the other processes of curing hernia.

